# Effect of a peer-educational intervention on provider knowledge and reported performance in family planning services: a cluster randomized trial

**DOI:** 10.1186/1472-6920-10-11

**Published:** 2010-02-02

**Authors:** Sakineh Mohammad-Alizadeh Charandabi, Rezagoli Vahidi, Lena Marions, Rolf Wahlström

**Affiliations:** 1 Division of Global Health (IHCAR), Department of Public Health Sciences, Karolinska Institutet, Stockholm, Sweden; 2 Nursing & Midwifery School, Tabriz University of Medical Sciences, Tabriz, Iran; 3 National Public Health Management Centre (NPMC), Tabriz, Iran; 4 Health & Nutrition School, Tabriz University of Medical Sciences, Tabriz, Iran; 5 Department of Women's and Children's Health, Karolinska Institutet, Stockholm, Sweden; 6 Department of Public Health and Caring Sciences, Uppsala University, Uppsala, Sweden

## Abstract

**Background:**

Peer education is an interactive method of teaching or learning which is widely used for educating school and college students, in a variety of different forms. However, there are few studies on its effectiveness for in-service education. The aim of this study was to evaluate the effect of an educational programme including peer discussions, based on a needs assessment, on the providers' knowledge and reported performance in family planning services.

**Methods:**

An educational programme was designed and applied in a random selection of half of in-charges of the 74 family health units (intervention group) in Tabriz at a regular monthly meeting. The other half constituted the control group. The programme included eight pages of written material and a two-hour, face-to-face discussion session with emphasis on the weak areas identified through a needs assessment questionnaire. The educated in-charges were requested to carry out a similar kind of programme with all peers at their health facilities within one month. All in-charges received one self-administered questionnaire containing knowledge questions one month after the in-charge education (follow-up I: 61 responses), and another one containing knowledge and self-reported performance questions 26 months later (follow-up II: 61 responses). Also, such tests were done for the peers facilitated by the in-charges one (105 responses) and 27 months (114 responses) after the peer discussions. Multiple linear regression was used for comparing mean total scores, and Chi square for comparing proportions between control and intervention groups, after defining facility as the unit of randomization.

**Results:**

The mean total percentage scores of knowledge (percent of maximal possible score) in the intervention group were significantly higher than in the control group, both at follow-up I (63%) and at follow-up II (57%); with a difference of 16 (95% CI: 11, 22) and 5 (95% CI: 0.4, 11) percentage units, respectively. Only two of the nine reported performance items were significantly different among the non in-charges in the intervention group at follow-up II.

**Conclusions:**

The educational programme including peer discussions using existing opportunities with no need for additional absence from the workplace might be a useful complement to formal large group education for the providers.

## Background

At the heart of each and every health system, the workforce is central to advancing health [1]. Its performance according to standards is the cornerstone of quality assurance in healthcare [2]. To perform well, it needs to have up-to-date knowledge. This is more essential today with rapid increases in knowledge and changing health care needs [1].

Each provider working at family health units in urban public health facilities in Iran covers identified households, offering first-level services, including all necessary maternal and child health care as well as family planning services. Inadequacy of continuous education was one of the barriers mentioned by the providers for high quality services in our previous study [3]. At present, most of their continuous education is in the form of 1-3 days courses conducted at district health centres, while there are no organized educational meetings at the facility level among the providers. Addressing all their educational needs by conventional methods alone seems difficult. It requires a lot of human and financial resources, and providers have to be away from their workplace for a long period of time, which can impede the quality of the services.

In 2004, the Iranian Ministry of Health and Medical Education published new national family planning guidelines in order to improve the quality of services offered [4]. In April 2005, most family health providers working at the public health facilities in Tabriz were educated about these guidelines during one-day meetings.

Experience shows that simply disseminating guidelines is ineffective for improving health staff competencies in care delivery [1]. Combining this with in-service education may be effective [5]. It has been shown that simple, low-cost approaches that follow the principles of interactive, close-to-practice training with adequate continuing support can be more effective than formal one-off, off-site training courses [1,5].

Peer education is an interactive method of teaching or learning which is widely used for educating school and college students, in a variety of different forms [6,7]. It has also been used for children [8], adolescents [9] and support groups, e.g. for clients with HIV/AIDS or chronic conditions like diabetes [10-12]. Many of the studies have shown that this method is as good as or even better than traditional education by teachers [6-9]. However, there are very few studies on its effectiveness for in-service education [13,14].

The objective of this study was to evaluate the feasibility and effectiveness of an educational method including peer discussions, on the providers' knowledge and reported performance in family planning services.

## Methods

### Setting

This study was conducted with staff working at the family health units of public health facilities in Tabriz, the capital city of East Azerbaijan province, with a population of around 1.4 million, located in the northwest of the country. All public family planning services were provided free of charge, at the front line by about 240 female midwives or family health technicians at family health units of 36 health centres (HCs) and 40 (two with only one staff member were excluded from the study) health posts (HPs) in the city. These two categories of staff provided combined oral contraceptives (COCs), progestin-only pills (POPs), depot medroxy-progesterone acetate (DMPA) and male condoms, and performed counselling and referrals for sterilization. However, copper intrauterine devices (Cu-IUDs, the only available IUD at the facilities) were exclusively inserted by the midwives. At each facility, one of the family health unit staff was in charge of the unit. The in-charges also provided services in the same way as the other staff members. Two one-day meetings were carried out at the district health centre for the in-charges, one day for the in-charges of health centres and one day for the health posts. In these meetings, the district health centre supervisors inform the in-charges about new guidelines or instructions. Using the meetings in order to educate the in-charges and having them disseminate the education to their peers at their workplaces seemed to be a suitable approach for addressing some of the providers' educational needs.

### Study design

All facilities with more than one staff member working at their family health unit and all the family health staff members providing front-line family planning services at those facilities were eligible to participate in the study.

The study was done in three phases. In phase one, a self-administered questionnaire, used to determine educational needs of the providers, was filled in by 64 in-charges attending a regular monthly meeting. Based on the results of phase one, an educational programme was designed and the questionnaire was modified to be used for the follow-up tests.

In phase two, half of the 74 eligible facilities were assigned to the intervention group by simple randomization among each type of the facilities (HCs or HPs) separately. The educational programme included eight pages of written material and an approximately two-hour face-to-face discussion session with more emphasis on the weak points identified in the needs assessment. It was applied for the in-charges of the intervention facilities (for the HCs and HPs separately) during the following regular monthly meetings, where 34 in-charges attended. Then these in-charges were requested to carry out a similar kind of programme with their peers. Their task was to distribute the written materials and to arrange at least one two-hourly peer discussion session/s on the content of the material at their health facilities within one month.

**Figure 1:**
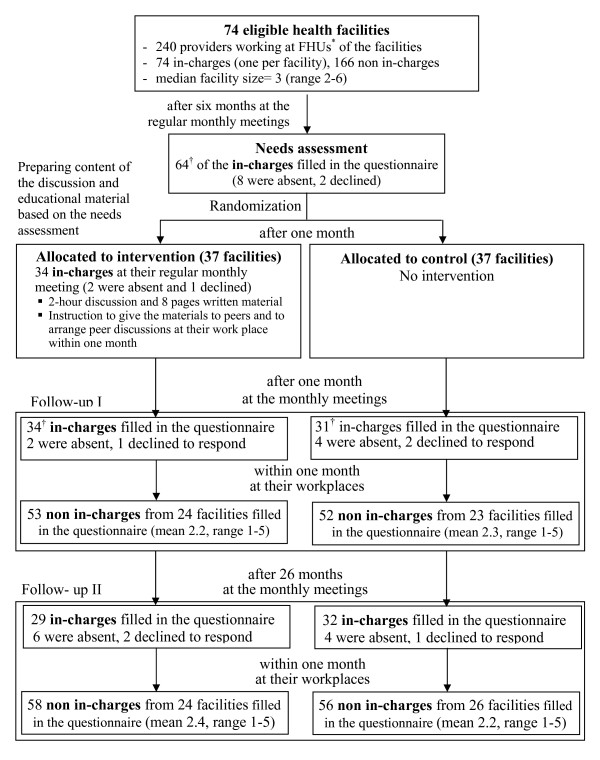
**Flowchart of the study design**.

In phase three, one (follow-up I) and 27 (follow-up II) months after the educational programme for the in-charges, follow-up tests were performed by all the in-charges attending at the monthly meetings. Such tests were also done by the peers at their workplace facilitated by the in-charges within one month after the in-charges' tests (Figure 1).

There was no special incentive for participation in this study. The in-charges were asked to participate by their supervisors, who have responsibility for their continuing education. In order to ensure the implementation of the educational program at the interventional health facilities, a written report signed by all participants had to be sent to the research team. There were also some questions about the quantity and quality of the educational sessions in the questionnaire at follow-up I.

Permission to undertake the study was obtained from the National Ethics Committee of the Ministry of Health and Medical Education in Tehran and from the Ethics committee at the Tabriz University of Medical Sciences. Approval was also received from authorities at the district health centre. Written informed consent was obtained from all participants.

### Data collection instruments

The questionnaire used for the needs assessment consisted of four parts; part 1: 26 general knowledge items answered with 'correct', 'incorrect' and 'don't know' options; part 2: Questions related to eligibility for using COCs, DMPA and Cu-IUD in 12 conditions answered with the following options (categorization according to the national guidelines): 'No contraindication', 'Relative contraindication', 'Absolute contraindication' and 'don't know'; part 3: five open-ended questions (missing three or more pills of COCs, need to re-take COC in case of vomiting, suitable time for removing IUD after last menstruation in women aged over 45, time period to use additional contraceptive method after vasectomy and condition for using breastfeeding as a contraceptive method) and part 4: eight and four questions respectively on the provider's own performance during IUD insertion and during DMPA injection.

To make the survey less lengthy and focusing on areas for change, seven items from part I and eight from part II, which were answered correctly by more than 80% of the participants at the needs assessments, were excluded at the post-tests. Also, two items from part I were excluded due to their unsuitability, as mentioned by the participants, and five questions from part 2 were excluded due to discrepancies between the national and WHO guidelines. The performance questions were excluded at follow-up I due to the short time (only one month) available to make any change of performance. Two items about the weak points of the providers' reported performance were added to part 1. Therefore, the post-test questionnaire consisted of three parts; 19 items in part 1 (general knowledge, see Annex), 22 questions in part 2 (eligibility of the methods), and five open ended questions (part 3). The same post-test questions plus the fourth part of the needs assessment questionnaire were used at follow-up II. The questionnaires also included some questions on characteristics of the providers, and at follow-up 1 there was also a question about the providers' views on the usefulness of the educational programme.

Content validity of the instrument was identified by the experts and reliability by Cronbach's alpha, separately for different parts, showing more than 0.7 in each part.

### Analysis

We calculated the proportion of correct responses (percent of maximal possible score) for each participant for each part of the questionnaire, assigning a score of 1 for each correct response and 0 for incorrect, 'don't know' or no answer; considering the same value for all items or questions. In the first and fifth open-ended question, all three requirements had to be answered correctly to get the score 1. The mean percentage of unanswered items or questions was less than 2% in part 1, less than 7.5% in part 2 and less than 9% in three of the open-ended questions in both groups and both follow-ups. Two of the open-ended questions, which had no 'don't know' option, were not answered by 20% and 40% of the participants. The mean of the percentage scores in each of the three parts, giving weight 1 to parts 1 and 2 and 0.5 to part 3, was considered the total percentage score of knowledge for each participant.

The performance items had five options (always, mostly, sometimes, seldom, never). We assigned a score of 1 for each 'always' or 'mostly' response in correct performance items and for 'never' or 'seldom' for incorrect performance items, and 0 for others, excluding no answers which were less than 3% in both groups.

Using Intercooled Stata 9 (Statasoft Inc, Tulsa, USA), Chi square was performed for comparing proportion of correct answers and appropriate reported performance in intervention and control groups, and multiple linear regression for identifying effects of the intervention on the mean percentage scores of knowledge and also in comparisons between follow-up I and II; adjusted by type of facility, age and degree of participants, and being an in-charge or not (see footnote in Table 2). Inter-class correlation coefficients within the facilities were between 0.1 and 0.4 for different parts of the questionnaire; 0.32 and 0.19 in the total percentage score of knowledge at follow-up I and II, respectively. Thus, all tests were performed after defining facility as the unit of randomization. Two-sided p < 0.05 was considered as a significant difference.

## Results

The characteristics of the participants in the intervention and control groups were similar at both follow-ups (Table 1).

**Table 1:** Comparison of characteristics of the providers in intervention (I) and control (C) groups

Characteristics	In-charges		Non in-charges		All providers	
	I	C	I	C	I	C
**Follow-up I**						
Degree, n (%)						
Midwifery (BSc*)	14 (45)	18 (62)	29 (58)	24 (46)	43 (54)	42 (51)
Midwifery(Tech.^†^)	4 (13)	4 (14)	9 (18)	12 (23)	13 (16)	16 (20)
Others	13 (42)	7 (24)	11 (22)	16 (31)	24 (30)	23 (28)
Type of facility, n (%)						
Health centre	16 (48)	17 (57)	31 (58)	32 (62)	47 (55)	49 (60)
Health post	17 (52)	13 (43)	22 (42)	20 (38)	39 (45)	33 (40)
						
Age, Mean ± SE	36.4 ± 1.1	35.1 ± 1.1	35.0 ± 1.1	32.5 ± 1.1	35.5 ± 0.8	33.5 ± 0.9

n^‡^	33	30	53	52	86	82

**Follow-up II**						
Degree, n (%)						
Midwifery (BSc)	18 (69)	21 (66)	24 (44)	22 (39)	42 (52)	43 (49)
Midwifery(Tech.)	2 (8)	3 (9)	11 (20)	17 (30)	13 (18)	20 (23)
Others	6 (23)	8 (25)	20 (36)	17 (30)	26 (32)	25 (28)
Type of facility, n (%)						
Health center	14 (48)	12 (38)	35 (60)	32 (57)	49 (56)	44 (50)
Health post	15 (52)	20 (62)	23 (40)	24 (43)	38 (44)	44 (50)
						
Age, Mean ± SE	33.2 ± 1.0	35.1 ± 0.8	34.1 ± 0.7	32.7 ± 1.4	33.8 ± 0.6	33.6 ± 0.9

n^‡^	29	32	58	56	87	88

* There were also 2 midwives with MSc degrees;

^† ^Technicians have two years of university education

^‡ ^Because of missing values, total numbers are not equal to the sums of the numbers for some characteristics

All in-charges (27 of 33 answered) and 89% (50 of 53 answered) of the non in-charges believed that such peer discussions were useful for their education to a high or very high degree.

The knowledge of the intervention group was significantly higher than that of the control group in regard to some of the knowledge items at both follow-ups. There were more significant differences at follow-up I than at follow-up II. At follow-up I, the percentages of correct answers to seven of the 19 items in the part 1 among non in-charges and to ten items among all providers in the intervention group were significantly higher than in the control group; while at follow-up II, the number of significantly different items was two and five, respectively [Additional file 1].

At follow-up I, the percentage scores of knowledge in part 1, 2 of the questionnaire, and in total in the intervention groups (both among non in-charges and all providers) were significantly higher than in the control groups. At follow-up II, there were significant differences between the groups in part 1 and total, but no significant differences in part 2. The differences were not significant in part 3 at the both follow-ups (Table 2).

Four of the in-charges of the intervention group facilities did not participate at the initial educational programme and six of those who participated did not carry out the peer discussions and only disseminated the educational material. Furthermore, some of the non in-charges did not participate at the meetings and only received the written material.

At follow-up I, 26 of non in-charges (15 from the intervention and 11 from the control group) reported getting only the printed materials. There were no significant differences between this group and those with no education in any parts and total percentage score of the knowledge [mean difference of the total percentage score was 3 (95% CI: -6,11) percentage points].

At follow-up II compared with follow-up I, the percentage scores of knowledge were significantly lower in part 1 [-12 (95% CI: -20, -3)] and in total [-7 (95% CI: -13, -1)] among the intervention group, but it was significantly higher in part 2 [8 (95% CI: 1, 14)] among the control groups. In other parts of the knowledge questions, the differences were not significant (Table 2).

**Table 2:** Mean (SD) and mean difference (95% CI) of percentage unit of knowledge scores* in intervention (I) and control (C) groups

Knowledge parts of the questionnaire	Non in-charges			All providers		
	I	C	Dif. (95% CI)^†^	I	C	Dif. (95% CI)
	Mean (SD)		Mean	Mean (SD)		Mean
**Follow-up I**						
Part 1: General (19 items)	75 (22)^†^	54 (17)	22 (11,34)	76 (21)	56 (16)	20 (12,29)
Part 2: Eligibility for using the methods (22 questions)	67 (22)	45 (17)	22 (12,33)	64 (20)	48 (16)	17 (10,24)
Part 3: Five open-ended questions	38 (28)	35 (24)	3 (-7,12)	39 (25)	32 (23)	6 (-0.5,13)
Total	64 (15)	46 (12)	18 (11,26)	63 (15)	48 (13)	16 (11,22)

n	53	52		86	82	

**Follow-up II**						
Part 1: General (19 items)	64 (20)	55 (14)	9 (3, 16)	64 (18)	59 (14)	6 (1, 11)
Part 2: Eligibility for using the methods (22 questions)	61 (22)	54 (18)	6 (-3, 12)	60 (20)	55 (17)	4 (-3, 12)
Part 3: Five open-ended questions	43 (23)	35 (24)	8 (-5, 22)	38 (23)	32 (22)	6 (-4, 15)
Total	59 (18)	51 (15)	8 (1, 15)	57 (16)	52 (13)	5 (0.4, 11)

n	58	56		87	88	

* These scores are useful for the objective of this study, but the knowledge level of the providers is probably underestimated, because items answered correctly by more than 80% in a pre-test had been excluded.

^† ^Difference (95% CI) of mean between intervention and control groups adjusted by type of facility (centre or post), and age (continuous variable), degree (MSc/BSC in midwifery or others) of participants, and being in-charge or not (only for comparing all providers).

At follow-up II, the percentages of correctly reported provider performance were significantly higher on two of nine items in the intervention group compared to the control group among the non in-charges and none among all the providers. On two other items among the non in-charges and on five items among the all providers, the percentages in the intervention group were 8-18 percentage points higher than in the control group, but the differences were not statistically significant (Table 3).

**Table 3:** Percentage of correctly reported provider performance* in the intervention (I) and control (C) groups before and 27 months (follow-up II) after the intervention

Items	Needs assess-ment^†^	Follow-up II			
		Non in-charges		All providers	
		I	C	I	C
**IUD insertion **(only for providers with at least five IUD insertions within a recent year)					
1. Recommend to use ibuprofen or other analgesics 30 minutes before insertion	35	65	61	66	58
2. Hand washing with soap and water before wearing gloves	54	76	58	71	59
3. Bimanual pelvic examination	55	65^‡^	33	57	44
4. Use of tenaculum	82	97^‡^	78	93	84
5. Use of a uterine sound	71	91	94	90	90
6. Inserting IUD with no-touch** method	---	67	66	65	67

n^††^	46	34	36	55	62

**DMPA injection **(only providers with at least five DMPA injection within recent one year)					
7. Hand washing with soap and water before injection	42	59	45	55	44
8. Massage injection site (Incorrect)	88	94	90	93	90
9. Recapping needles after injection (Incorrect)	52	80	80	76	78

n	58	51	51	80	81

* is done 'always' or 'mostly' in correct and 'never' or 'seldom' in incorrect items

^† ^The needs assessment was done only for the in-charges

^‡ ^p < 0.05 compared with the control group

** This question was excluded from the needs assessment analysis because the meaning of this method was not clear to some participants; it was explained in the follow-up questionnaire

^†† ^IUD could only be inserted by the midwives

## Discussion

The total scores of provider knowledge in the intervention group were significantly higher than in the control group at both follow-ups, but the difference between the groups at the follow-up II was less than at follow-up I. This may indicate a reduced effect of the intervention by time and a need to reinforce the education. Another possible reason is high provider turn-over, resulting in dilution of the effect. More than every third provider at the time of follow-up II was new compared to the situation at the time of the needs assessment. There was also some contamination between the groups, i.e., a few providers from the intervention facilities moved to the control group or the other way around. The other possible reason is that it could be the effect of other interventions in this area during these two years; for example, on eligibility for using the method where there were no longer any differences between the intervention and control groups at follow-up II, and there were significant differences between the control groups at follow-up I and II. These reasons may also have caused the lack of any significant effect on the providers' performance in most items. On the other hand, this relatively limited short intervention may not have been enough to change provider performance. Moreover, in this study, we did not allocate any credit points for the programme, which otherwise might have been useful in terms of encouraging the providers to more actively participate in such programmes and increasing their effectiveness.

In all analyses on the effectiveness of the interventional programme, we included all providers working at the facilities in their assigned groups without considering their actual participation in the educational programme. Some of the providers in the intervention group did not get the education or only received the written material, which would not have been effective in improving their knowledge, based on our results. All of these circumstances may have caused a dilution of the effect of the programme.

The positive effects of the intervention may seem relatively high for such a limited intervention. There is no obvious cultural or environmental factor that can be considered an explanatory factor for a better outcome in this context. At the time of the study, some quality promotion programs, like quality monitoring and conducting counseling educational workshop, including client rights, had been implemented in the facilities. Although this took place in both the intervention and control facilities, the providers in the intervention facilities may have been more receptive to the type of quality improvement method used in the study than they would have been otherwise. It might be a factor to consider when drawing conclusions about how this method can be used in other contexts.

For ethical reasons, in this study, all questionnaires were filled in anonymously and coded at the facility level. The research team had no access to these codes and could therefore not compare the individual responses before and after intervention, and not use statistical tests based on paired data. Randomization was done after the needs assessment; it was therefore not possible to directly assess similarity between the intervention and control groups regarding their knowledge and performance before intervention. In order to address this limitation we compared results of follow-up I in the in-charges' control group with the needs assessment results on the common items.

The mean score of the in-charges in the control group in part one of follow-up I was significantly higher [11 (95% CI: 4, 18) percentage points] than the needs assessment while there were no significant differences in parts two and three (-1 and 2 percentage points difference, respectively). It might indicate that the basic knowledge of the control group was not less than that of the intervention group, and any significant increase in knowledge scores in the intervention group compared with the control group could then be considered as related to the intervention. A possible reason for the significant increase in part 1 of knowledge in the control group may be an increased emphasis placed on the teaching by the district supervisors, after getting the results of that part one week after the needs assessment.

We also compared some regular statistics about contraceptive services and supplies provided for clients during three months, prior to the intervention between the intervention and control group facilities. We found that there were no significant differences between the groups with regard to mix of contraceptive methods, number of different type of contraceptives distributed, and the proportion of high-risk women (women aged less than 18 or more than 35, with three children or more, or with a less than two years old child) who used modern contraceptives.

We could not calculate the sample size as we did not know the design effect of the cluster randomization, and therefore recruited all eligible facilities and providers in the study. However, after the study, the calculated statistical power for the total percentage score of knowledge was 81 and 90 per cent for follow-up I and II, respectively; considering m1 = 50, m2 = 60, sd1 = sd2 = 17, α = 0.05, and a design effect due to cluster sample allocation for follow-up I estimated as 1.74 and for follow-up II as 1.44.

The results of this study of effects of the educational intervention on provider knowledge confirm the positive effect of peer-based learning for in-service education, which has been shown in the few other studies [14,15] done in this area. However, consequent effects on provider performance are not conclusive in our study, as we only have one long-term assessment based on reported performance. More studies in this area with more rigid design are needed, especially in terms of its effects on provider performance. It would be an advantage to assess such effects using more valid measures like unannounced standardized patients [16,17] or vignettes [18,19].

## Conclusions

This type of educational programme including peer discussions, using existing resources with no need for people to take off from work might be a useful complement to formal large group educational efforts for providers. Providing opportunities for reinforcing the education may be needed to maintain and strengthen the effect of such programmes.

## Competing interests

The authors declare that they have no competing interests.

## Authors' contributions

SMAC conceived the study; participated in its design, developed and implemented the educational programme, collected the data, analyzed the data, and drafted and finalized the manuscript. All co-authors participated in design of the study and developing the educational programme, analyzing, rewriting draft versions and critically revising the manuscript. All authors have read and approved the final manuscript.

## Pre-publication history

The pre-publication history for this paper can be accessed here:

http://www.biomedcentral.com/1472-6920/10/11/prepub

## Supplementary Material

Additional file 1 **Answers to the items in part 1 of the questionnaire**. Percentage of right answers by providers to the items in part 1 of the questionnaire in intervention (I) and control (C) groups. Click here for file
