# Supplementary material for: Effect of a peer-educational intervention on provider knowledge and reported performance in family planning services: a cluster randomized trial
Source: BMC Med Educ. 2010 Feb 2;10:11. doi: 10.1186/1472-6920-10-11 (PMC2830222; doi:10.1186/1472-6920-10-11)
Supplement: Additional file 1 — Answers to the items in part 1 of the questionnaire. Percentage of right answers by providers to the items in part 1 of the questionnaire in intervention (I) and control (C) groups. [file 1472-6920-10-11-S1.DOC]

**Additional file**. Percentage of right answers by providers to the items in part 1 of the questionnaire in intervention (I) and control (C) groups

| **Items** | **Needs**  **Assess-ment^*^** | **Follow-up I** | | | | **Follow-up II** | | | |
| --- | --- | --- | --- | --- | --- | --- | --- | --- | --- |
|  |  | **Non in-charges** | | **All providers** | | **Non in-charges** | | **All providers** | |
|  |  | **I** | **C** | **I** | **C** | **I** | **C** | **I** | **C** |
| 1. Married women under the age of 15 don't need to use contraceptives if their regular menstrual cycles have not yet started. (incorrect) | 53 | 89 | 77 | 89^†^ | 76 | 84^†^ | 57 | 85^†^ | 67 |
| 1. After taking emergency contraceptive pills (ECPs), the woman’s monthly period sometimes starts a few days later than expected. (correct) | 73 | 77 | 65 | 80 | 67 | 86 | 75 | 86 | 76 |
| 1. Taking ECPs should be started from the morning of the next day after unprotected intercourse. (incorrect) | 56 | 83 | 69 | 79 | 68 | 66 | 68 | 71 | 74 |
| 1. Taking ECPs cannot protect the woman against pregnancy the days after taking them. (correct) | 52 | 75 | 71 | 74 | 73 | 26 | 43 | 40 | 30 |
| 1. If ECPs fail, the risk of fetal abnormality is not increased. (correct) | 60 | 83 | 67 | 83 | 72 | 71 | 64 | 68 | 70 |
| 1. ECPs usually prevent pregnancy by prevention of implantation. (incorrect) | 10 | 53^†^ | 13 | 43^†^ | 15 | 29 | 18 | 21 | 16 |
| 1. Almost all COC users experience at least one of their side effects. (incorrect) | 15 | 58^†^ | 31 | 58^†^ | 27 | 38 | 23 | 31 | 20 |
| 1. A woman who uses an IUD has to avoid from doing heavy work. (incorrect) | 37 | 68^†^ | 31 | 74^†^ | 30 | 57^†^ | 30 | 59^†^ | 41 |
| 1. If pregnancy continues without removing the IUD, the risk of serious infections, which could threaten the woman’s life, may increase. (correct) | 45 | 70 | 46 | 70 | 52 | 50 | 55 | 52 | 50 |
| 1. If pregnancy continues without removing IUD, risk of fetal abnormality can be increased. (incorrect) | 69 | 92^†^ | 60 | 87^†^ | 67 | 71 | 61 | 72 | 68 |
| 1. IUD should be inserted later in women after caesarean section than in women after normal delivery. (incorrect) | 29 | 72^†^ | 44 | 77^†^ | 52 | 57 | 41 | 60 | 53 |
| 1. A suitable antibiotic must be given after IUD insertion to prevent infection. (incorrect) | 48 | 68^†^ | 21 | 69^†^ | 35 | 59 | 38 | 61^†^ | 42 |
| 1. Using DMPA may cause infertility. (incorrect) | 60 | 77 | 73 | 74 | 74 | 74 | 77 | 77 | 82 |
| 1. Injection site should be massaged after injection of DMPA to facilitate good absorption. (incorrect) ^‡^ | --- | 79**^†^** | 46 | 83^†^ | 54 | 97 | 89 | 95 | 92 |
| 1. After injection of DMPA, needles should be recapped. (incorrect) ^‡^ | --- | 64 | 46 | 70 | 54 | 86 | 82 | 83 | 83 |
| 1. If female sterilization is not done in the first week of postpartum, it must be delayed until 42 days postpartum. `(correct) | 77 | 83 | 67 | 83 | 71 | 72 | 75 | 69 | 74 |
| 1. Major changes in bleeding pattern will occur after female sterilization. (incorrect) | 48 | 83 | 63 | 85**^†^** | 60 | 69 | 50 | 66**^†^** | 50 |
| 1. If pregnancy occurs after female sterilization, it is more likely to be ectopic than in a woman who uses no contraception. (correct) | 61 | 89 | 83 | 83 | 78 | 66 | 63 | 68 | 66 |
| 1. A condom makes sex less enjoyable for almost all couples. (incorrect) | 35 | 64 | 48 | 74**^†^** | 45 | 55 | 45 | 63**^†^** | 48 |
| n | 64 | 53 | 52 | 87 | 82 | 58 | 56 | 87 | 88 |

^*^ The needs assessment was done only for the in-charges, ^†^ Compared with control group p<0.05

^‡^ These items were added to follow-up questionnaires
